# Fast algorithms for computing phylogenetic divergence time

**DOI:** 10.1186/s12859-017-1916-1

**Published:** 2017-12-06

**Authors:** Ralph W. Crosby, Tiffani L. Williams

**Affiliations:** 1 Department of Computer Science, College of Charleston, Charleston, SC USA; 2 Department of Computer Science, Northeastern University, Charlotte, NC USA

**Keywords:** Phylogenetics, MCMC, Divergence time

## Abstract

**Background:**

The inference of species divergence time is a key step in most phylogenetic studies. Methods have been available for the last ten years to perform the inference, but the performance of the methods does not yet scale well to studies with hundreds of taxa and thousands of DNA base pairs. For example a study of 349 primate taxa was estimated to require over 9 months of processing time. In this work, we present a new algorithm, AncestralAge, that significantly improves the performance of the divergence time process.

**Results:**

As part of AncestralAge, we demonstrate a new method for the computation of phylogenetic likelihood and our experiments show a 90% improvement in likelihood computation time on the aforementioned dataset of 349 primates taxa with over 60,000 DNA base pairs. Additionally, we show that our new method for the computation of the Bayesian prior on node ages reduces the running time for this computation on the 349 taxa dataset by 99%.

**Conclusion:**

Through the use of these new algorithms we open up the ability to perform divergence time inference on large phylogenetic studies.

## Background

Darwin envisioned the relationship between all the various species as a great tree with living species as the leaves and branches leading downward to extinct ancestors. A recent estimate places the number of living species at 8.8 million ±1.3 million [1]. While projects such as the Open Tree of Life (http://opentreeoflife.org) seek to develop an all-encompassing tree, the vast majority of phylogenetic analysis focus on a particular branch of the tree. In addition to knowing how species are related to each other (as shown by the tree topology), we would also like to know when these species diverged from their ancestors. Adding dates to historical events allows us to temporally connect events and thereby draw additional conclusions from the data.

**Fig. 1:**
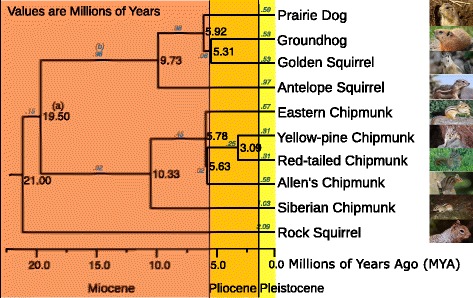
Divergence Time of the Ground Squirrels. The annotations on each branch refer to the length of the branch calibrated to millions of years. For example, the node marked with a star shows that the prairie dogs diverged from the groundhog/golden squirrel linage 5.92 million years ago. Geological era’s are shown to allow for correlation of species divergence to geological events

Consider the ground squirrels as represented by the tribe Marmotini (Fig. 1). The divergence times computed are shown in units of Millions of Years [2]. These times closely approximate the most recent published divergence time data for the family [3]. It is apparent that there was a significant increase in the species of ground squirrels during the late Miocene to early Pliocene eras. It is also known that there was a large increase in savannas and grasslands worldwide during the same period [4]. It is therefore possible to hypothesize an expansion in habitat fostering an expansion in ground dwelling mammals like the Marmotini. The addition of divergence time data allowed for exploration of correlations between these evolutionary events. Dates allow evolutionary events to be compared not only with other evolutionary events (like the expansion of grasslands) but with geological and historical events.

If divergence time provides helpful information, why isn’t it always done as part of a phylogenetic analysis? An informal survey of recent phylogenetic studies by the authors showed that less than half of the studies that included more than 100 taxa also included the determination of divergence time. Divergence time is the last step in a long process; the time from the start of sample collection to a published tree can easily be years.

But, divergence time inference itself can be a long process. Modern methods for the computation of phylogenetic divergence time are based on the determination of the Bayesian posterior probability. This probability is the product of four components, the likelihood of the tree, the Bayesian prior probability of the ancestral node ages, the Bayesian prior of the rates of evolution along each branch of the tree and the Bayesian prior probability of the evolutionary model (and other “nusiance” parameters). To compute the actual posterior probability this product is normalized by the probability of the data. Iterative, Markov chain Monte Carlo methods are used to determine the shape of the posterior distribution and eliminate the need to compute the generally intractable probability of the data.

For the well-regarded MCMCTree program [5], the time complexity for the computation in a single MCMC step is composed of the following components, repeated for each of the *n*−1 inner nodes:

- The traversal of the species tree building the age list: $\mathcal {O}(n)$.
- A traversal of the species tree computing the density of the calibration nodes: $\mathcal {O}(n)$.
- Sorting the age list: $\mathcal {O}(n\ \text {log}\ n)$.
- Traversing the sorted age list computing the density of the non-calibration nodes: $\mathcal {O}(n)$.

giving an overall complexity of (*n*−1)(*n* log*n*) or $\mathcal {O}(n^{2}\ \text {log}\ n)$.

Our experimental analysis of the MCMCTree program [5] has shown that on a primate dataset consisting of 349 taxa [6] over 91% of the total elapsed time is used in the computation of the likelihood. Of the remaining 9% time, approximately half is computation of the prior of the node ages. More specifically, a two week run time to compute the divergence time using MCMCTree’s approximated likelihood algorithm would be required for the primate data set. Estimates of the run time of MCMCTree’s exact likelihood algorithm were on the order of over two years of execution time for the primate data.

### Our contributions

We introduce a new approach; AncestralAge, which significantly reduces the time required to compute phylogenetic divergence time. Our contributions fall under the following three categories.

- *Subtree site compression algorithm.*
  The likelihood of a tree and it’s associated parameters (e.g. ancestral node ages) refer to the probability that a set of parameters were responsible for the set of taxa observed today. The computation of this value is typically the most expensive single component of divergence time inference (or any phylogenetic inference for that matter). There have been a number of different approaches to reducing the cost of the likelihood computation including algorithmic improvements [7, 8], approximation methods [9], and parallelization of the process [10–12], and [13]. These approaches have been focused on the problem in the context of phylogenetic inference wherein the topology of the tree is being inferred along with the branch (edge) lengths. In the context of divergence time inference where the tree topology is fixed, there has been far less research [14].
  We have developed a new algorithm, subtree site compression, for the computation of phylogenetic likelihood that reduces the time required for an *exact likelihood* computation by over 90%. This method is similar to that of Kobert, et al. [15] but further improves the performance through the use of a hash table to maintain the lookup instead of the fixed table with a fairly complex least-recently-used algorithm of Kobert et al. The use of the hash table reduces the time to both insert into the table and access the table to $\mathcal {O}(1)$. Furthermore, we extensively analyze the run time varying key parameters (e.g. number of sites).
- *Prior of Ages algorithm.*
  The Bayesian prior on the ages of the nodes in the tree ties the fossil calibrations into the statistical model. There has been some discussion of the use of other, non-Bayesian methods, for the computation of divergence time [16] but incorporation of fossil data into these models has not been successfully accomplished. The use of a Bayesian prior for fossil calibrations is a natural and easy way to incorporate calibration information into the model. The fossils are actually prior knowledge about the model.
  We demonstrate a new algorithm for the computation of the prior of node ages that reduces what had been a time complexity of $\mathcal {O}(n^{2}\ \text {lg}\ n)$ to best and worst case complexities of $\mathcal {O}(n)$ and $\mathcal {O}(n^{2})$ respectively.
- *Extensive experimentation using the AncestralAge and MCMCTree approaches.* In addition to MCMCTree, Beast [17] is actively used for computing divergence times. Beast performs both phylogenetic inference and divergence time inference as a single step. It is possible to specify a starting tree for the MCMC process to Beast but the topology of the tree is considered a model parameter and potentially perturbed throughout the process.
  Since MCMCTree is intended for divergence time only, its statistical methods have been used as the basis for the algorithms in AncestralAge. We compare the subtree site compression and prior of ages algorithm in AncestralAge with MCMCTree in terms of accuracy of results and running time on a variety of biological and synthetic datasets. While MCMCTree computes exact and approximated likelihood, AncestralAge computes the exact likelihood. Our results show a reduction of 97% in elapsed time on the aforementioned primate dataset over the MCMCTree exact likelihood algorithm and a 28% reduction in elapsed time compared to the approximated likelihood algorithm in MCMCTree. Thus, our experimental results show that our exact likelihood computation is often much faster than both MCMCTree’s exact and approximated likelihood computations.

## Our subtree site compression algorithm

### Motivation

**Fig. 2:**
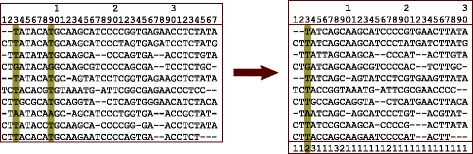
Full Site Compression. Sites (columns) 3 and 9 have identical values for all taxa and therefore are compressed into a single column with a count of 2

Most phylogenetic and divergence time programs support a simple compression technique wherein the sites in the alignment are examined and duplicate sites are compressed. Each site in the alignment has a counter added to indicate the number of copies of the site found. During the summation of the logs of the site likelihoods, the log value is multiplied by the counter to generate the equivalent of repeatedly computing the same value for the multiple copies of the site. In Fig. 2, sites 3 and 9, for example, have identical values for every taxa and therefore the counter for that pattern is set to 2.

This original site compression technique provides some improvement in the overall performance. For example, the 61,249 sites in the 79 genes included in the primates dataset [6] compress to 32,789 unique sites (47% compression). The problem is that as the number of taxa in an alignment increases, the probability of finding duplicate sites naturally tends to decrease. For the primates dataset, as expected, the highest compression ratios appeared in the genes with the fewest taxa.

Our insight for the subtree site compression algorithm was that if the topology of the tree is fixed, as is the case in divergence time inference, a similar approach could be taken to the compression of subtrees. Consider an alignment containing only a pair of the taxa from a larger tree. If two different sites in this two taxa alignment have identical values, they will produce the same likelihood vectors and transition probability matrices (TPMs) since the edge lengths will be the same. Therefore, there is no need to repeat the computation for any subsequent site containing the same pattern for those two taxa. This approach, subtree site compression, can be applied to every subtree in the gene.

**Fig. 3:**
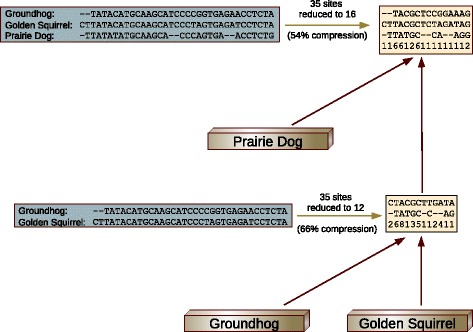
Subtree Site Compression. At the lowest level, the sequence alignment for the Groundhog and the Golden Squirrel compresses to 12 sites. At the next level, 35 sites compress to to 16 sites

As an example, consider two of the taxa from our Marmots study, the Golden Squirrel and the Groundhog. In Fig. 3, the two sequences for these species are shown along with with the results of applying subtree site compression. The 35 sites in the alignment compress to 12 unique combinations, a 66% reduction. Going up another level in the tree, if the Prairie Dog is added to the alignment and subtree compression performed again, the 35 sites are reduced to 16 sites, a 54% reduction.

Using subtree site compression, the maximum number of combinations appearing in the compressed set at any inner node is min(5^*n*^,*s*), where *n* is the number of leaves in the subtree and *s* is the length of the original alignment. Obviously 5^*n*^ quickly exceeds even large numbers of sites, but every inner node whose children are both leaves will have a maximum of (*c*+1)^2^, where *c* is the number of codes (e.g., c = 4 for DNA). One more than the number of codes is used to allow for the “unknown” or missing data code. In a balanced tree, *n*/2 of the *n*−1 inner nodes will satisfy this condition.

### Algorithm description

The algorithm for subtree site compression adds two additional entities at each inner node in the tree; a hash table and a site lookup table. The hash table is used to determine whether, as the subtree alignment is scanned, the code combination has been seen before. The site lookup table is used to index into the likelihood vectors and TPMs for the descendants of the node.

At a given inner node in the tree, the sites corresponding to an alignment of only those leaves that are descendants of the node are considered one at a time. The concatenation of the code values for the leaves at the site is used as the key into the hash table. If the key already exists in the hash table, no further processing is done. If the key does not exist in the hash table, it is added and an entry is appended to the site lookup table. This index of the new entry in the site lookup table is set as the value pointed to by the hash table entry.

The site lookup table entry contains two fields, one for each of the descendants of the node. These fields provide the indices into the descendants likelihood vectors or TPMs. To compute the likelihood for an alignment position on an inner node, the site lookup table entries for the position are used to get the index into the descendants likelihood vector (if an inner node) or the descendants TPM (if a leaf node). If the descendant is a leaf node, the index points to the row in the descendants TPM corresponding to the value of the site in the leaf. If the descendant is an inner node, a key is constructed containing the site values for only those leaves that are under the descendant. This key is then used to access the descendant node’s hash table and retrieve the index value in the descendants likelihood table.

At the root node there is one additional field in the site lookup table, the repeat count as in the original site compression algorithm.

### Example

**Fig. 4:**
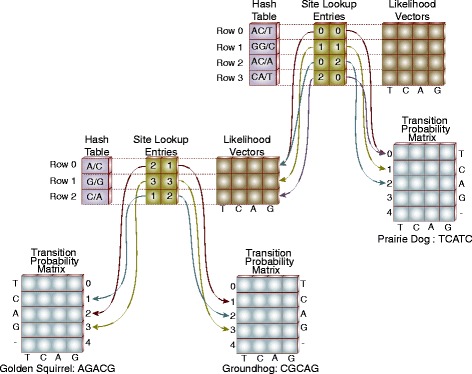
Subtree Site Compression Algorithm. The structures supporting the likelihood calculation for a set of three species is shown. At the lower level, the ancestor of the Golden Squirrel and the Groundhog has three unique site combinations shown as three entries in the hash, site lookup and likelihood vectors. The ancestor of all three species has four unique combinations in it’s alignment. The entries in the site lookup table pointing to the descendant of the Golden Squirrel and the Groundhog contain indicies into the likelihood vectors for the descendant

If we start at the Golden Squirrel and Groundhog in Fig. 4 their ancestor will have a hash table, site lookup table, and likelihood vector. An alignment of five columns of the sequences from each of the taxa is shown. Out of these five sites, there are three unique combinations: *AC*, *GG*, and *CA*; therefore the hash table will only contain those three entries. The first row in the site lookup table will point to the “A” row in the TPM for the Golden Squirrel and the “C” row in the TPM for the Groundhog. Similarly the second and third rows in the site lookup table will point to the appropriate rows in the descendants TPMs.

For the next level up in the tree, the ancestor of three species; the Golden Squirrel, the Groundhog and the Prairie Dog is shown. In this case, the four entries in the hash table correspond to the four unique values appearing the alignment of the three species. The first site lookup table row contains an index into the likelihood vector for the Golden Squirrel and Groundhog’s descendant vector corresponding to that portion of the key associated with the Golden Squirrel and Groundhog (*AC*). The other half of the first site lookup table row contains the index of the “T” row in the Prairie Dog’s TPM.

### Algorithm analysis

At any inner node in a tree, subtree site compression is limited by two factors: the total length of the sequence alignment and the number of leaves in the subtree. In the worst case, the number of rows in the likelihood vector at a subtree site compressed node will be the number of codes plus one (to account for the “unknown” or “missing” code value) taken to the power of the number of leaves. For example, in the case of an inner node whose children are both leaves using the four DNA codes, the most number of rows that will exist in the likelihood vector is (4+1)^2^=25. The maximum number of rows in any given likelihood vector for a DNA coded alignment is therefore the minimum of the sequence alignment length, *s*, and 5^*n*^ where *n* is the number of taxa in the subtree.

Given the exponential growth in the maximum number (5^*n*^) associated with the leaf count, the maximum will quickly become limited by the sequence alignment length with performance no worse than the existing site compression method. But, in a balanced tree, *n*/2 out of the *n*−1 total inner nodes will have two children and in these cases the leaf count exponent will, in all likelihood, be significantly smaller than the sequence alignment length. Using the DNA code set, even with three or four leaves there is a good chance that the maximum for the exponential of the number of leaves (5^*n*^) would be less than the maximum for a typical sequence alignment.

At the other extreme, in a completely unbalanced, “caterpillar”, tree, the sequence length would quickly dominate the worst case and the impact of subtree site compression would be limited to the lowest one (5^2^), two (5^3^) or possibly three (5^4^) nodes.

## Our prior of ages algorithm

The Bayesian prior on the ages of the nodes in the tree ties the fossil calibrations into the statistical model. The use of a Bayesian prior for fossil calibrations is a natural way of incorporating the calibration information in the model.

### Statistical model

Our algorithm implements the statistical model of Yang [18]. Following the usage in Eqs. 4 and 10 in Yang 2006 [18]), *g*(*t*) is the probability distribution function (pdf) value for the age of a node under the birth-death-sampling (BDS) model used and *G*(*t*) is the cumulative distribution function (cdf) value for the age of a node under the BDS model.

Equation (1) is a reformulation of Yang’s Eq. 11 for the marginal density of the calibration nodes.

1 $$  f_{BDS}(\mathbf{t}_{c}| t_{R}, n) = \frac{(n-2)!}{\prod_{1}^{c} h(i)} \prod_{1}^{C} G^{\prime} (i)  $$

where

2 $$  h(i) = \left\{\begin{array}{ll} (\mathbf{R}_{i} - 1)! & i = 0 \\ (\mathbf{R}_{i} - \mathbf{R}_{i-1} - 1)! & 0 < i < c \\ (n-2-\mathbf{R}_{i-1})! & i=c \end{array}\right.  $$

and

3 $$  G^{\prime}(i) = \left\{\begin{array}{ll} G(t_{i})^{\mathbf{R}_{i} - 1} & i = 0\\ \left(G(t_{i}) - G(t_{i-1})\right)^{\mathbf{R}_{i}-\mathbf{R}_{i-1}-1} & 0 < i < c \\ \left(1 - G(t_{i-1})\right)^{n-2-\mathbf{R}_{i}} & i = c \end{array}\right.  $$

**R** defines a list containing the rankings of the ages of all *c* calibration nodes among the *n*−2 node ages.

By expanding and canceling terms, the conditional density of the non-calibration nodes given the calibration nodes can then be calculated as follows.

4 $$\begin{array}{*{20}l}  f\left(\mathbf{t}_{\bar{c}}|\mathbf{t}_{c}\right) &= \prod_{i=1, i \not\in c}^{s-2} g(t_{i}) \frac{\prod_{i=0}^{c} h(i)}{ \prod_{i=0}^{c} G^{\prime}(i)} \end{array} $$

In practice, this is computed using the logs of the values.

5 $$\begin{array}{*{20}l}  \ln f\left(\mathbf{t}_{\bar{c}}|\mathbf{t}_{c}\right) &= \sum\limits_{i=1, i \not\in c}^{s-2} \ln g(t_{i}) + \sum\limits_{i=0}^{c} \ln h(i) - \sum\limits_{i=0}^{c} \ln G^{\prime}(i) \end{array} $$

### Algorithm description

In the MCMCTree implementation of the model, each time a new age is proposed, a list of all node ages is generated and sorted. This list is then traversed and, for each entry in the list, the appropriate values (*g*(*t*) or *G*(*t*)) are computed depending on whether the node is a calibration or a non-calibration node. This calculation occurs in it’s entirety for each new age proposed.

**Fig. 5:**
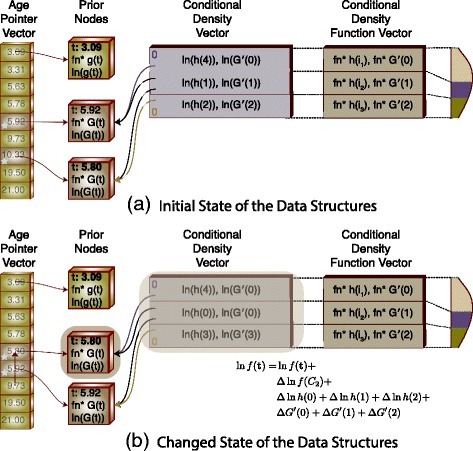
Change in the Age and Position of a Calibration Node. In this example the age of the first calibration is changed such that the ordering of the calibration PNs is changed. In this case all CDV entries that reference either of the calibration PNs require recomputation

**Fig. 6:**
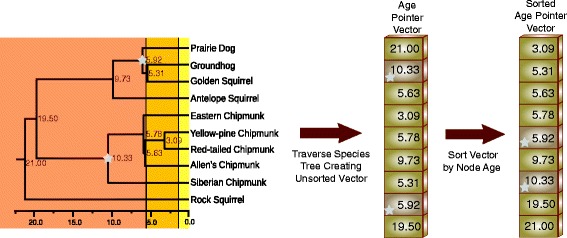
Building the Age Pointer Vector. During a depth-first traversal of the species tree, the ages of the nodes are appended to the vector. Entries associated with calibration nodes are marked as such and the vector is sorted by age

The key to our new prior algorithm is a set of data structures that allow intermediate values to be retained across computations. These structures are shown in Fig. 5 a. Each non-leaf node in the species tree will have an associated prior node (PN). For the order statistics, it is necessary to construct a list, sorted by age, of all nodes. In Fig. 6, we demonstrate the process of traversing the tree, building a list of pointers to the PNs, and then sorting this list to create the Age Pointer Vector (APV). By maintaining this list as a vector, it is possible to compute the rank value by subtracting indices into the vector without the requirement to traverse a list. The APV is created once during model initialization.

A reference to the parameter containing the current age of the node along with the index of the node in the APV is held in each PN instance. A non-calibration subclass extends this information with the addition of a pointer to the function responsible for computing the *g*(*t*) value along with the log of the current *g*(*t*) function value.

A pair of vectors is associated with the segments of the birth-death-sampling PDF. Each segment will have an entry in the conditional density vector (CDV) as well as an entry in the conditional density function vector (CDFV). Each CDV entry will contain the current values of the *h*(*i*) and *G* ^′^(*i*) functions (the second and third terms in Eq. (5)) associated with the segment. The CDV entry will also hold pointers to the starting and ending prior node instances. A CDFV entry is associated with but independent of a CDV entry as it’s information is static throughout the execution of the model while the CDV entry contents are volatile. The CDFV entry contains pointers to the functions used to compute the *h*(*i*) and *G* ^′^(*i*) values for the segment. In reality, these functions are functional objects (functors) that are initialized depending on the position of the CDFV entry in the CDV list. For example, the first *h*(*i*) CDFV entry will always compute it’s function value with the knowledge that it’s the first segment. This is particularly important for the first and last CDFV entries as their computations differ from the computations for “middle” nodes (see Eqs. (2) and (3)).

Computation of the prior is handled as transactions against the data structures with the goal being minimization of the computation required for any individual transaction. A new proposed age for a node in the species tree triggers the transactions. Transactions are categorized depending on whether the node with the age proposal holds a fossil calibration.

1. *A change in the date of a non-calibration node that does not affect the ordering of the APV.* In this case, none of the rankings of the calibration nodes change and therefore there is no change to any of the values in the CDV. The new value for the prior can be computed as the old value updated with the change in the *g*(*t*) value associated with the single non-calibration node.
  6 $$ \ln f(t) = \ln f(t) + \Delta ln g(t)  $$
2. *A change in the date of a non-calibration node that changes the ordering of the APV.* In this case, the new node age is either younger or older than one or more nodes in the APV. If the movement of the node does not alter the ranking of the calibration nodes, the order of the entries in the APV is changed. The remainder of the transaction is handled the same as for the previously discussed change. In other words, the new node age did not change the position of any calibration nodes in the APV.
  If the new node age does cause a change in the position of one or more calibration nodes, the contents of both CDV entries that border on the changed calibration node(s) need to be recomputed.
  7 $$\begin{array}{*{20}l} \ln f(t) = &\ln f(t) +  \\ &\Delta \ln g(t) +  \\ &\Delta \ln h(i-1) + \Delta \ln h(i) +  \\ &\Delta G^{\prime}(i-1) + \Delta G^{\prime}(i) \end{array} $$
3. *A change in the date of a calibration node that does not change the ordering of the APV.* The PDF value for the new calibration date is computed whenever a new date is proposed for a calibration node. In the case where the ordering of the APV is not changed, the new value for the calibration nodes *G*(*t*) function is computed and the values for the *G* ^′^(*i*) values for the two CDV entries that refer to the calibration node are recalculated. Note that the *h*(*i*) functions do not require recalculation since the ranking of the nodes has not changed.
  8 $$\begin{array}{*{20}l} \ln f(t) = &\ln f(t) +  \\ &\Delta \ln f(t_{PN}) +  \\ &\Delta G^{\prime}(i-1) + \Delta G^{\prime}(i) \end{array} $$
  The *G* ^′^(*i*) value as shown in Eq. (3) is computed using the difference between the CDF values for the bordering CDV segments. This value is raised to the power associated with the number of non-calibration nodes associated with the segment. Since, in this case, only one of the CDF values has changed, the change in the log of the *G* ^′^(*i*) can be computed as
  9 $$ \Delta G^{\prime}(i) = (rank_{i-1} - rank_{i}) (\ln G^{\prime}_{new}(i) - \ln G^{\prime}_{old}(i))  $$
  requiring only one computation of the CDF.
4. *A change in the date of a calibration node that changes the ordering of the APV.* As with any change to a calibration node, the PDF value for the new date is computed. A change to the position of a calibration node in the APV will by definition change the ranking for at least that node. This will require recalculation of the two CDV nodes that border the node. In this case the rankings of the nodes have changed and both the *h*(*i*) and *G* ^′^(*i*) values will need to be recomputed.
  10 $$\begin{array}{*{20}l} \ln f(t) = &\ln f(t) +  \\ &\Delta \ln f(t_{PN}) +  \\ &\Delta \ln h(i-1) + \Delta \ln h(i) +  \\ &\Delta G^{\prime}(i-1) + \Delta G^{\prime}(i) \end{array} $$
  As shown in Fig. 5, if the change in the ordering of the APV is such that the node moves past one or more calibration nodes, the set of CDV entries requiring recomputation will increase to encompass any entries whose borders have changed.

### Algorithm analysis

New values for the prior are only required when a new node age is proposed and therefore only computed *n*−2 times for each MCMC iteration. The computational complexity per MCMC step is not excessive either at $\mathcal {O}({n^{2}\ \text {log}\ n})$. The problem is that the constant multiplier for the computation is large. A significant amount of computation is required for each node in the tree.

For our algorithm, the complexity is related to the distance up or down the list a node moves as the result of a new age proposal. Since the data structures are only adjusted ad not created during each MCMC step, there is no computational cost associated with the list generation or sort during MCMC processing.

In the worst case, it is theoretically possible that an age proposal could cause a calibration node to move from one end of the APV to the other. If all inner nodes had calibrations associated with them, the result would be the recalculation of *n*+1 total CDV entries for a worst case complexity of $\mathcal {O}(n)$ for one age parameter proposal and $\mathcal {O}(n^{2})$ for an MCMC step.

In practice, this is extremely unlikely for two reasons. First, the step size used for age proposals is small. If a step size were used that caused nodes to move large distances within the APV, the MCMC process itself would most likely be unstable and probably never reach stationarity. Second, the number of nodes with calibrations tends to be a small percentage of the overall nodes (4% in the case of the primates dataset) so that the length of the CDV is small relative to the total number of inner nodes (*n*−1).

The best case complexity for a single age parameter proposal is simply $\mathcal {O}(1)$ and for an MCMC step $\mathcal {O}(n)$. If there is no movement in the APV, only a single calculation of *g*(*t*) or *G*(*t*) and 2 associated CDV entries would be required for a single age parameter calculation.

In terms of space complexity there is some additional memory required but no change in the actual complexity. There is a prior node and an entry in the APV for each inner node (including the root) in the species tree to give a space complexity of $\mathcal {O}(n)$ for these structures. For the CDV and CDFV, the number of entries is equal to one more than the number of calibrations. Since the number of calibration nodes cannot exceed the number of non-leaf nodes *c*≤*n*, the worst space space complexity for these structures will also be $\mathcal {O}(n)$ giving a total complexity of $\mathcal {O}(n)$ for all the structures.

## Methods

### Biological datasets

Below are the four biological datasets studied in this paper. The MCMCTree and AncestralAge parameters for total steps, burn-in, and number of samples for each dataset are shown in Table 1.

**Table 1:** MCMC Parameters for the experimental datasets. In this table, the MCMC parameters for the four sample datasets are shown

	Monkeys	Squirrels	Influenza	Primates
Total MCMC steps	42,000	110,000	110,000	110,000
Burnin MCMC steps	2,000	10,000	10,000	10,000
Samples taken	20,000	5,000	20,000	5,000

- *Monkeys.* Provided as an example in the PAML distribution and was extensively analyzed by Yang, et al. [18]. There are 7 taxa in the tree with 9993 DNA sites in three genes.
- *Squirrels.* Consists of 69 taxa with 7248 total DNA sites in 5 genes. Analyzed by the authors [2].
- *Influenza.* Provided as an example in the PAML distribution and was also extensively analyzed by Yang, et al. [19]. There are 289 viral taxa in the tree with 1710 DNA sites in one gene.
- *Primates.* Consists of 349 taxa with 61,249 total DNA site in 79 genes [6]. Estimates of the run time for MCMCTree using its approximated likelihood algorithm were in excess of 14 days. Estimates of the run time for the exact likelihood algorithm were on the order of over two years of execution time. Comparisons of the inferred ages for this dataset between AncestralAge and MCMCTree were not performed since the approximated likelihood algorithm of MCMCTree and the exact likelihood algorithm of AncestralAge are not computing dates in the same way—potentially leading to unfair comparisons.

### Synthetic datasets

To understand the scaling characteristics of AncestralAge, datasets of synthetic trees with varying characteristics were generated. Trees were generated with varying number of taxa (*t*∈{20..200}) and used as input to the Seq-Gen program [20] to produce DNA sequences with lengths varying from 10,000 to 100,000 sites across 10 to 100 genes. All sequences were generated using the HKY [21] evolutionary model with a transition/transversion ratio, *κ*, of 2 and gamma variation among sites using 4 discrete categories.

All datasets were processed by AncestralAge. The small tree for each set was also processed using MCMCTree. Attempts to process all the generated trees with MCMCTree were unsuccessful due to excessive execution times. For MCMCTree, experiments were run using the exact likelihood model. The same evolutionary model (HKY) parameters and rate model (independent) were used for both programs. Each test was allowed to run for 100 MCMC steps. This value was chosen since, in the case of the exact likelihood method of MCMCTree, the MCMC step times were large and the goal of the experiments was only to determine an average step time. We hypothesized that 100 MCMC steps would be sufficient to overcome the impact of any initialization and termination. In all cases the CPU time required for initialization and termination outside of the MCMC process itself was less than 1% of the total CPU. Performance information was obtained through API calls to the Linux kernel as well as the through the PAPI performance library [22].

### Computational platform

All tests were run on the Texas A&M University Brazos high performance cluster (http://brazos.tamu.edu). Each node on the cluster consists of dual 2.5 GHz Intel quad core processors and 32 GB of memory.

### Reporting computational time

Values reported are the average times required for a single MCMC step.

## Results

In this section, we provide an analysis of the AncestralAge from both the perspective of the dates inferred and the performance of the algorithms.

### Divergence time validation

Three biological datasets (Monkeys, Squirrels, and Influenza) were used to validate the model. As explained in the Experimental Methodology section, the primates dataset was too large to be run by MCMCTree. The datasets were run with AncestralAge and MCMCTree using the same parameters (evolutionary model, multiple sequence alignment, input tree, and prior hyperparameters). MCMCTree supports an approximated and exact likelihood whereas AncestralAge computes exact likelihood. Thus, model validation is based on an exact likelihood computation in order to compare the dates returned by the two approaches.

For each branch *b* in the tree, let *d*_1_ and *d*_2_ represent the date produced on that branch by MCMCTree and AncestralAge, respectively. Differences in dates between the two programs were normalized by the mean of the two dates (*d*_1_ and *d*_2_) giving an indication of the relative difference, *d*=2(*d*_1_−*d*_2_)/(*d*_1_+*d*_2_). In all cases, the results from AncestralAge were within *ε*=0.005 (.995<*d*<1.005) of the results returned by MCMCTree. Thus, the dates returned by AncestralAge were within the 95% credibility interval returned by MCMCTree.

### Performance analysis

Table 2 presents the results of running AncestralAge and MCMCTree on the validation and primates datasets. As the size, in terms of taxa, genes and sequence length, increased it can be seen that the performance of AncestralAge continued to improve relative to MCMCTree. For the monkey dataset, MCMCTree outperformed AncestralAge in both the approximated and exact likelihood tests. But, in all the larger datasets, AncestralAge outperformed MCMCTree with exact likelihood. On the squirrel dataset, AncestralAge outperformed MCMCTree with exact likelihood by a factor of 14.7. This increased to factors of 38.4 and 37.3 for the influenza and the primates datasets respectively. For the model validation datasets, MCMCTree with approximated likelihood outperformed AncestralAge but the difference narrowed as the dataset size increased, from a factor of 35.4 for the monkey dataset to a factor of 3.8 for the influenza dataset. When the problem size was increased to the 349 taxa and 79 genes of the primates dataset, AncestralAge outperformed MCMCTree approximated likelihood by a factor of 1.4.

**Table 2:** Ancestral age performance on sample datasets. Performance using the exact and approximated likelihood algorithms in MCMCTree is compared with AncestralAge using the three sample datasets as well as the Primates dataset. Times shown are the average times for a single MCMC step

	Monkeys	Squirrels	Influenza	Primates
Total sequence length	9993	7248	1710	61249
Taxa	7	69	289	349
Genes	3	5	1	79
Statistical parameters	51	563	869	51,483
MCMCTree exact likelihood	0.00065sec/step	1.029sec/step	20.942sec/step	167.000sec/step
MCMCTree approximated likelihood	0.00007sec/step	0.012sec/step	0.140sec/step	6.266sec/step
AncestralAge	0.00230sec/step	0.070sec/step	0.545sec/step	4.473sec/step

We hypothesize that for the monkeys dataset, the number of parameters was so small (12) that the time for AncestralAge was dominated by task dispatching associated with our multi-threaded implementation. For all other experiments the subtree compressed likelihood provided a significant performance advantage over MCMCTree with exact likelihood. We further hypothesize that as the problem size (number of taxa in particular) increased, the percentage of the run time associated with the likelihood computation in both AncestralAge and MCMCTree with approximated likelihood decreased. This allowed the percentage of the run time associated with the prior of ages to increase. Once the problem size became large enough, the performance advantage of our prior of ages algorithm allowed AncestralAge to outperform MCMCTree with approximated likelihood.

To further understand the performance characteristics of AncestralAge relative to MCMCTree a set of synthetic data was generated. Of particular interest was the determination of the point at which AncestralAge performance would exceed the performance of MCMCTree with approximated likelihood. But, errors were encountered in the generation of the gradient and Hessian matrix required for the MCMCTree approximated likelihood method so only results for MCMCTree with exact likelihood are presented. Further research will be required to understand why the synthetic datasets produced errors in the generation of the gradient and Hessian matrix for MCMCTree.

#### Varying numbers of genes

Trees with *l*∈{10,20...100} genes were generated. Each gene had independent data but a constant sequence length of 2000 sites.

**Fig. 7:**
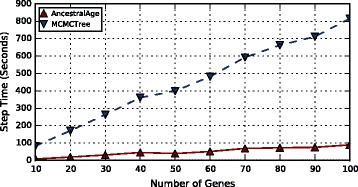
CPU Times for Varying Numbers of Genes. The number of genes, *l*∈{10,20...100}, was varied for a constant sequence length of 2000 sites per gene. Each tree was run with both MCMCTree (exact likelihood algorithm) and AncestralAge. The number of taxa was held constant at 100. The times displayed are the average for a single MCMC step

The results of the experiments are shown in Fig. 7. The performance of AncestralAge increases in a nearly linear fashion with the number of genes. Times for MCMCTree with exact likelihood also increased in a linear fashion with a much greater slope. The speedup for AncestralAge over MCMCTree was fairly consistent with a mean speedup of 9.0.

#### Varying sequence lengths

Trees with varying DNA sequence lengths, *s*∈{1000,2000...10000}, sites per gene were generated. Each tree was composed of 100 taxa and 10 genes.

**Fig. 8:**
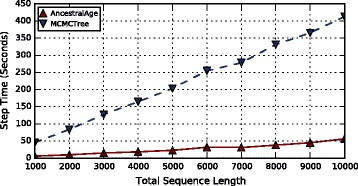
CPU Times for Varying DNA Sequence Lengths. The total num- ber of sites across 10 genes was varied from 10000 to 100000 in increments of 1000. Each tree was run with both MCMCTree (exact likelihood algo- rithm) and AncestralAge. The number of taxa was held constant at 100. The times displayed are the average for a single MCMC step

The results of the experiments are shown in Fig. 8. It can be seen that the performance of AncestralAge is scaling with the overall sequence length. Times for MCMCTree with exact likelihood also increased in a linear fashion with a much greater slope. The speedup for AncestralAge over MCMCTree was also fairly consistent with a mean speedup of 8.5.

#### Varying numbers of taxa

Trees with varying numbers of taxa, *n*∈{20,40...200}, were generated. Each tree composed of 10 genes of 200 sites each.

**Fig. 9:**
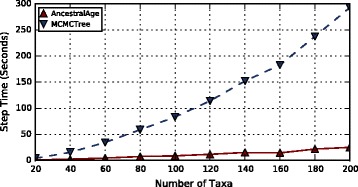
Step CPU Times for Varying Numbers of Taxa. The number of taxa n, was varied from 20 to 200 in increments of 20. Ten genes of 2000 sites each were generated for each taxa. Each tree was run with both MCMCTree (exact likelihood algorithm) and AncestralAge. The times displayed are the average for a single MCMC step

The results of the experiments are shown in Fig. 9. It can be seen that the performance of AncestralAge is scaling in a nearly linear fashion with the number of taxa. Times for MCMCTree with exact likelihood appear to be increasing at a better than linear rate. In fact the speedup for AncestralAge over MCMCTree increased from a factor of 4.3 for the 20 taxa experiment to a factor of 11.6 for the 200 taxa experiment.

### Impact on the primates dataset

Using the primates data, we will compare the performance of the existing site compression algorithm from MCMCTree with our subtree site compression algorithm.

There are a total 10,792 inner nodes in the 79 genes with a total sequence length of 61,249 sites compressed to 32,789 unique sites. Factoring in the individual sequence length for each gene, a total of 4,427,618 site likelihood calculations will be performed to fully compute the likelihood for the species tree. With an average of 32 floating point operations (flops) per site likelihood calculation, a total of 141,683,776 flops will be performed to compute the likelihood for the tree.

### Subtree site compression

The number of leaf edge calculations (*n*) will be roughly equal to the number of inner node edge calculations, *n*−2, so the average flops to compute the likelihood at a particular node and site will be considered the average of the leaf (4) and inner node (60) values; 32. Using this value the calculation of likelihood across the entire tree will require 141,683,776 flops. Running on a modern desktop machine (3.0 GHz Intel Core i7) this has been experimentally shown to require, on average, 0.109 s.

For the subtree site algorithm, the 10,792 inner nodes in the 79 genes compressed to a total of 438,138 positions in the various likelihood vectors for this same number of site likelihood computations. Using the same average flops for a calculation the total flops to compute the tree likelihoods is only 14,020,416. Experimentally, the time required for this computation, using the same hardware as before, is 0.011 s, a 90.1% reduction over the site compression alone thereby experimentally validating the calculations.

### Prior of ages

Experimental analysis of 110,000 MCMC steps over the primates dataset showed that in 73% of the new age proposals no positional changes occurred in the Age Point Vector (APV) and of the remaining 27% of the proposals only 4 proposals (out of a total of 3,480,000 age proposals) moved a node (calibration or non-calibration) more than one slot in the APV. This shows that, in reality, the performance of our new algorithm is much closer to $\mathcal {O}(n)$ than $\mathcal {O}(n^{2})$, as would be expected on biological data.

## Conclusions

Phylogenetic divergence time has proven to be a rich area for computational research. When we started our work with the MCMCTree program it became apparent that this program had been developed as a test platform for statistical theory relating to divergence time. The other significant application, Beast, took a different approach to the problem hypothesizing that combining phylogenetic inference with divergence time inference would produce a better model. In some small cases, this has been proven [23] and [24], but this combination also limits the ability of the biologist to independently estimate the phylogenetic tree and the dates for the nodes of the tree.

We found that neither Beast or MCMCTree scaled to the hundreds of trees and thousands of base pairs of DNA that are being used in modern studies. In order to provide a framework for further research into the process of phylogenetic divergence time, we developed the AncestralAge framework. This framework has facilitated research into new algorithms in a number of areas relating to divergence time including likelihood computation and Bayesian prior computation.

In order to improve the efficiency and performance of the dating process we first focused on developing new algorithms for dating individual trees. Our subtree site compression algorithm for the computation of phylogenetic likelihood has demonstrated a 90.1% improvement over existing methods. Our incremental prior of ages algorithm has shown a better than 100x improvement over the comparable computation in MCMCTree.

We believe our new algorithms will allow researchers to perform divergence time inference on datasets of hundreds to thousands taxa with millions of DNA sites.
